# Three-dimensional versus two-dimensional high-definition laparoscopy in cholecystectomy: a prospective randomized controlled study

**DOI:** 10.1007/s00464-019-06666-5

**Published:** 2019-02-01

**Authors:** Hanna Koppatz, Jukka Harju, Jukka Sirén, Panu Mentula, Tom Scheinin, Ville Sallinen

**Affiliations:** 1 Department of Abdominal Surgery, University of Helsinki and HUS Helsinki University Hospital, Haartmaninkatu 4, 00029 HUS Helsinki, Finland; 2 Department of Transplantation and Liver Surgery, University of Helsinki and HUS Helsinki University Hospital, Helsinki, Finland

**Keywords:** Stereopsis, Gall bladder, Laparoscopic, 3D, Visual, LCC

## Abstract

**Background:**

While 3D laparoscopy increases surgical performance under laboratory conditions, it is unclear whether it improves outcomes in real clinical scenarios. The aim of this trial was to determine whether the 3D laparoscopy can enhance surgical efficacy in laparoscopic cholecystectomy (LCC).

**Method:**

This prospective randomized controlled study was conducted between February 2015 and April 2017 in a day case unit of an academic teaching hospital. Patients scheduled for elective LCC were assessed for eligibility. The exclusion criteria were: (1) planned secondary operation in addition to LCC, (2) predicted to be high-risk for conversion, and (3) surgeons with less than five previous 3D laparoscopic procedures. Patients were operated on by 12 residents and 3 attendings. The primary endpoint was operation time. All surgeons were tested for stereoaquity (Randot® stereotest). The study was registered in ClinicalTrials.gov (NCT02357589).

**Results:**

A total of 210 patients were randomized; 105 to 3D laparoscopy and 104 to 2D laparoscopy. Median operation time as similar in the 3D and 2D laparoscopy groups (49 min vs. 48 min, *p* = 0.703). Operation times were similar in subgroup analyses for surgeon’s sex (male vs. female), surgeon’s status (resident vs. attending), surgeon’s stereovision (stereopsis 10 vs. less than 10), surgeon’s experience (performed 200 LCCs or below versus over 200 LCCs), or patient’s BMI (≤ 25 vs. 25–30 vs. > 30). No differences in intra- or postoperative complications were noted between the 3D and 2D groups.

**Conclusion:**

3D laparoscopy did not show any advantages over 2D laparoscopy in LCC.

Laparoscopy has become the gold standard for many abdominal operations, and the spectrum is increasing all the time with the inclusion of more and more complex operations [1–3]. The technological evolution has been tremendous, however, since the beginning of the laparoscopic era, the lack of depth in operative field visualization has been a limitation. A three-dimensional (3D) operative field is visualized on a two-dimensional (2D) screen, which needs to be converted back to a 3D field in the surgeon’s brain. The first laparoscopes with 3D capability were introduced in the 1990s, but enthusiasm quickly subsided as they caused disturbing side-effects in surgeons such as visual strain and headache [4].

Recently, modern high-definition 3D laparoscopes have emerged and are used in many hospitals. Although they make visual depth available, these systems have several negative aspects as well. First, the laparoscopes are 10 mm in size, compared to the possibility to use 5 mm 2D laparoscopes, and 10 mm 3D laparoscopes might thus cause potential risk for port site hernias. On the other hand, atleast one 5 mm port site needs to be enlarged to extract the specimen. Second, specific glasses need to be worn for the duration of the operation, and the 3D monitor is more prone to visual disturbances if the positioning is not optimal. Third, 3D laparoscopes are more expensive than their 2D counterparts and thus this economic burden needs to be justified if it is to be applied routinely in clinical practice.

Several experimental studies have shown benefit of 3D in terms of operative times, but circumstances are not equivalent to clinical settings [5].

Laparoscopic cholecystectomy (LCC) is one of the most common procedure in general surgery and is usually carried out as a day case surgery. A few randomized trials have tried to evaluate the benefits of 3D laparoscopy in LCC, but the conclusions are limited by the small number of patients included in the trials.

The aim of this study was to compare the surgical efficacy and safety of 3D versus 2D laparoscopy in LCC.

## Materials and methods

This was a randomized controlled trial conducted in the day surgery department of an academic teaching hospital functioning also as a secondary referral center (HUS Helsinki University Hospital). Patients scheduled for elective LCC in an operating room equipped with 3D laparoscopic instrumentation were assessed for eligibility. Patients could be included if they were scheduled for an elective LCC for symptomatic cholecystolithiasis. The exclusion criteria were: (1) planned secondary operation in addition to LCC, (2) predicted to be high-risk for conversion to laparotomy (such as history of numerous abdominal operations, peritonitis, or acute cholecystitis within the previous 3 months), and (3) surgeons with inadequate experience in 3D laparoscopy (defined as less than five 3D laparoscopic procedures). The set limit used to determine adequate experience for the 3D procedures was based on an earlier report indicating that the learning curve for 3D laparoscopy included five procedures [6].

### Power calculation and randomization

The primary outcome measure was operation time. Secondary outcome measures included conversion rate, intraoperative complications, postoperative complications (Clavien–Dindo), need for hospital stay, estimated blood loss, hospital readmission, mortality, and operation room time. For power calculation purposes, the operation time for LCC procedures performed in 2013 in the department were extracted from the electronic operating room scheduling board. The cases for inclusion in this calculation were searched using ICD-10 code JKA21 as the primary procedure in the year 2013. Cases with a secondary procedure code were excluded. The mean operative time for LCC was 56.5 min [standard deviation (SD) 25.5 min, *n* = 521]. Standard deviations were assumed to be similar in the forthcoming 3D procedures. Based on these figures, 80% power, 0.05 alpha, 1:1 allocation, and two-tailed power analyses were performed. The study was powered to detect 10 min differences in operative procedures by including 208 patients. A block randomization with a 1:1 allocation and a randomly varied block size of 4 to 6 was generated using Blockrand 1.1 package with R Statistical Software. Randomization cards were enclosed in sequentially numbered, opaque-sealed envelopes. At the time of inclusion, the envelopes were opened sequentially by the operating surgeon prior to the operation. Patients were blinded to their randomization group.

### Instrumentation and interventions

Wolf® (Richard Wolf Medical Instruments®, Chicago, Illinois, USA) 2D/3D laparoscopic HD device with a non-deflectable 30$^\circ$ scopes were used for all the operations. The system can display both 3D and 2D images. For the 3D group cases, the device was set to 3D, and for the 2D group it was set to 2D. The surgeons were allowed to switch from 3D to 2D if needed, (e.g., during trocar insertion) but switching from 2D to 3D in the 2D group was not allowed. Adherence to the randomized group (2D or 3D) was assessed by a case report form, which the surgeon filled after the operation. In the 3D group, the surgeons and an assistant standing on the patients left side wore passive polarizing glasses through the whole operation, whereas no extra glasses were worn in the 2D cases. Both the surgeon and the assistant used the same monitor, which was located on the patients' right side. The surgeons were allowed to define the proper viewing position for themselves to avoid any disturbances in vision.

Residents performing the operations had at least 3 years surgical experience and were on rotation at the day case surgery unit. Residents, if deemed proficient, were allowed to perform LCC procedures independently. The number of previous procedures (LCC or 3D procedures in general) were recorded for each surgeon (classified as < 50, > 50, or > 200 previous cases for LCC; < 10, > 10, or > 50 previous cases for 3D procedures in general). The subjective satisfaction of each surgeon was collected based on a 0–10 Likert scale score, and the surgeons were free to express comments or concerns regarding the laparoscope in free-text form after the operation. The stereo acuity was measured using the Randot® Stereotest (Stereo Optical, Chicago, Illinois, USA), but surgeons were neither selected nor excluded based on the test. The Randot test consists of ten sets of three circles, one of which has a crossed disparity and appears to be closer. Between the sets the disparity decreases from 400 to 20 s of arc. If the surgeon could not distinguish the differences between the sets, he/she was considered stereo blind (0 points). Otherwise the level of stereopsis was defined as the last circle identified correctly. The level of perfect stereopsis was defined to 20 s of arc (10 points).

The operation time was defined as the time from the first incision until closure of the skin. A standard LCC in this study included the insertion of four trocars, one 12-mm trocar inserted supraumbilically, one 10-mm trocar inserted in the epigastric region, and two 5-mm trocars inserted in the right flank. Routinely, the fundus of the gallbladder was grasped, Calot’s triangle was dissected revealing the critical view of safety, and the gallbladder was detached from the liver using a monopolar hook. The gallbladder was extracted through the epigastric incision. After extraction, the abdominal cavity was checked to ensure hemostasis. Drains were not used. Surgeons were allowed to deviate from these routines if deemed necessary for patient safety.

Thirty-day complications were assessed from the electronic medical records, and the patients were contacted by phone at 30 days after the operation. At that time, post-discharge complications were assessed. In cases where the patient did not respond to phone calls, they were contacted by letter.

### Statistical analysis

The statistical analyses were performed using SPSS® version 22 (IBM, Armonk, NY). Continuous variables were compared between groups using the Mann–Whitney *U* test and *p* values < 0.05 were considered statistically significant. Subgroup analyses based on the sex of the surgeon, the surgeon’s level of experience, resident versus attending status, and stereovision were specified a priori, and additionally subgroup analysis was performed for patients’ body mass index (BMI).

This study was approved by the institutional review board and the ethical board of Helsinki University Hospital. All patients gave informed written consent to participate the study. The study was registered in ClinicalTrials.gov before randomization (NCT02357589).

## Results

**Fig. 1:**
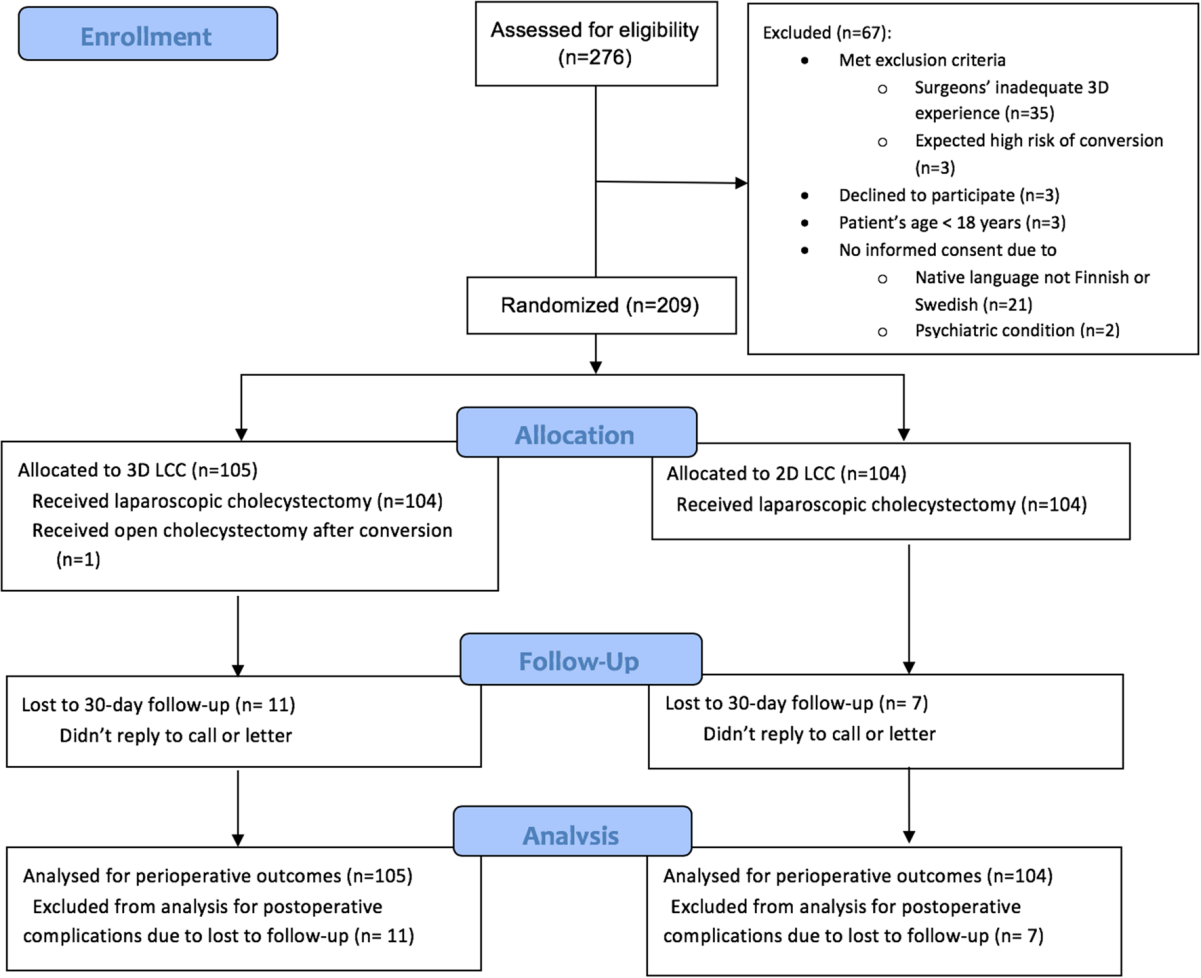
CONSORT flow-chart of patient selection, randomization, and follow-up

A total of 276 patients were assessed for eligibility beginning February 2015. Randomization to the LCC trial reached 209 patients by April 2017 (Fig. 1).

There were no differences in the patients’ basic characteristics between the 3D and 2D groups (Table 1). The majority had no other health issues. Patients were operated on by 12 residents and 3 attendings with variable experience in laparoscopic cholecystectomy (Table 1). Two surgeons (13%) were stereo blind. One operation was converted from 3D to 2D due to dysfunction of the light cable.

**Table 1:** Basic characteristic of patients undergoing laparoscopic cholecystectomy and of the surgeons operating on them *N* (%)

	3D (*n* = 105)	2D (*n* = 104)
Age, median; IqR	48.5; 56.4–24.7	49.9; 38.2–59.3
BMI, median; IqR	28.4; 31.9–25.1	27.3; 31.0–24.7
Male patient	31 (29.5%)	27 (26.0%)
ASA-classification		
1	37 (35.2%)	47 (45.2%)
2	60 (57.1%)	49 (47.1%)
3	8 (7.6%)	8 (7.7%)
Charlson's comorbidity index		
0	97 (92.4%)	100 (96.2%)
1	6 (5.7%)	2 (1.9%)
2	2 (1.9%)	1 (1.0%)
3	0	1 (1.0%)
History of earlier abdominal operation(s)		
Open	4 (3.8%)	3 (2.9%)
Laparoscopic	9 (8.6%)	12 (11.5%)
Indication		
Symptomatic gallbladder stones	100 (95.2%)	98 (94.2%)
Earlier pancreatitis and gallbladder stones	1 (1.0%)	0
Gallbladder and bile duct stones	2 (1.9%)	0
Gallbladder polyp	2 (1.9%)	2 (1.9%)
Other	0 (1.0%)	2 (1.9%)
Earlier cholecystitis	0	2 (1.9%)
Surgeon		
Man	89 (84.8%)	94 (90.4%)
Attending	66 (62.9%)	64 (61.5%)
Resident	39 (37.1%)	40 (38.5%)
Surgeon experience in LCC, cases		
< 50	2 (1.9%)	4 (3.8%)
50–200	46 (43.8%)	37 (35.6%)
> 200	57 (54.3%)	63 (60.6%)
Surgeon experience in 3D laparoscopy, cases		
5–10	19 (18.1%)	14 (13.5%)
10–50	53 (50.5%)	58 (55.8%)
> 50	33 (31.4%)	32 (30.8%)
Surgeon stereo acuity, stereopsis 10	74 (70.5%)	80 (76.9%)

*2D* two-dimensional, *3D* three-dimensional, *ASA* The American Society of Anesthesiologists physical status classification,* BMI* body mass index, *IqR* interquartile range, *LCC* laparoscopic cholecystectomy

One 3D laparoscopy was converted to open cholecystectomy due to chronic cholecystitis. There were no conversion to open surgery in the 2D laparoscopy group. No differences were noted in intraoperative or postoperative complications (Table 2). All postoperative complications were Clavien–Dindo class 1 or 2, with no differences between the 3D and 2D groups. Three patients (1.6%; *n* = 3 in 3D, *n* = 0 in 2D) were readmitted and 38 (19.9%; *n* = 17 in 3D, *n* = 21 in 2D) had an outpatient visit within 30 days.

**Table 2:** Outcome measures after laparoscopic cholecystectomy

	3D *n* = 105	2D *n* = 104	*p* value
Operating room time, minutes, median; IqR	112.0; 100.0–128.0	108.5; 96.3–124.3	0.261
Operation time, minutes, median; IqR	49.0; 39.0–65.0	48.0; 38.5–59.8	0.703
Estimated blood loss, ml, mean (SD)	4.8 (13.3)	11.9 (60.9)	0.245
Intraoperative complications			
None	91 (86.7%)	89 (85.4%)	0.843
Gallbladder rupture	7 (6.7%)	10 (9.7%)	
Intraoperative bleeding, minor	4 (3.8%)	4 (3.9%)	
Bleeding, liver bed, haemostat needed	2 (1.9%)	1 (1.0%)	
Iatrogenic lesion (liver cyst)	1 (1.0%)	0	
Postoperative complication*			
Total	21 (22.3%)	22 (22.7%)	0.731
Clavien–Dindo I	15 (16.0%)	12 (12.4%)	0.537
Abnormal pain	9 (9.6%)	3 (3.2%)	
Urinary retention	2 (1.9%)	0	
Bleeding (abdominal wall)	0	3 (3.2%)	
Postoperative fever, (liver bed hematoma)	1 (1.0%)	0	
Dizziness	0	1 (1.0%)	
Nausea	0	1 (1.0%)	
Gastroenteritis	0	1 (1.0%)	
Respiratory infection	0	1 (1.0%)	
Other	3 (3.2%)	2 (1.9%)	
Clavien–Dindo II	6 (6.4%)	10 (10.3%)	0.435
Surgical wound infection	6 (6.4%)	7 (7.2%)	
Thrombophlebitis from iv canula	0	2 (1.9%)	
Vaginal candidiasis	0	1 (1.0%)	
Satisfaction with laparoscopic view, attendings, median; IqR	10; 10	10; 9–10	0.259
Satisfaction with laparoscopic view, residents, median, IqR	9; 8–10	8; 7–8	< 0.001

*IqR* interquatile range, *LCC* laparoscopic cholecystectomy, *SD* standard deviation

*Five patients had more than one complication

None of the patients died within 90 days after the operation. Although scheduled in a day case surgical unit, 20 patients (19.0%) in the 3D arm and 17 patients (16.3%) in the 2D arm required an overnight stay in hospital mostly due to social issues.

The time spent in the operating room and operation time were similar in 3D and 2D arms (Table 2). There were no differences in operation times within the subgroup analysis based on the sex of the surgeon, surgeon’s level of experience, status (resident / attending), stereovision, or patient’s body mass index (Table 3). Attendings had similar satisfaction in 3D and 2D groups, but residents preferred 3D laparoscopy (Table 2).

**Table 3:** Subgroup analysis of operation time for laparoscopic cholecystectomy

Subgroup	3D minutes; IqR (N)	2D minutes; IqR (N)	*p* value
Surgeon status			
Attendings	42.5; 35.0–53.0 (66)	42.0; 33.3–50.0 (64)	0.406
Residents	62.0; 49.0–79.0(39)	60.0; 47.0–84.0 (40)	0.596
Sex*			
Male resident	54.0; 44.0–81.0 (25)	60.0; 45.8–84.0 (30)	0.375
Female resident	69.0; 51.5–75.5 (14)	66.5; 48.8–91.3 (10)	1.00
Stereovision*			
Stereopsis 10	52.0; 49.0–73.0 (15)	50.0; 43.5–64.5 (17)	0.602
Stereopsis ≤ 9	68.0; 45.5–82.0 (24)	74.0; 52.0–95.0 (23)	0.209
3D experience			
≤ 50	50.0; 39.0–67.8 (72)	50.0; 40.3–67.3 (72)	0.867
> 50	47.0; 37.0–55.0 (33)	45.0; 35.3–50.8 (32)	0.412
LCC experience			
≤ 200	53.5; 42.0–72.5 (48)	60.0; 45.5–84.0 (41)	0.120
> 200	44.0; 36.5–55.0 (57)	42.0; 34.0–50.0 (63)	0.167
Patient BMI			
≤ 25	42.5; 36.0–52.5 (24)	43.0; 33.0–52.0 (31)	0.845
25–30	50.0; 38.5–66.8 (42)	47.0; 40.0–60.5 (40)	0.590
> 30	50.0; 47.0–72.0 (39)	55.0; 46.5–74.0 (33)	0.354

*2D* two-dimensional, *3D* three-dimensional, *BMI* body mass index, *IqR* interquartile range, *LCC* laparoscopic cholecystectomy

*Residents

## Discussion

Clinical studies regarding 3D laparoscopy are scarce with low numbers of patients and surgeons. We conducted a prospective randomized controlled study to clarify the role of 3D laparoscopy in terms of surgical efficacy and safety during one of the most common laparoscopic operation, LCC. To our knowledge, this was the largest study evaluating 3D on LCC. However, we did not find any benefit of 3D laparoscopy in performing LCC.

The first clinical report comparing 3D and 2D laparoscopy was a small randomized trial with 30 patients in each group that underwent LCC and were operated on by four experienced surgeons [4]. Similar to our results, no differences in operating times was found. Contrary to our results, the surgeons were dissatisfied with 3D laparoscopy complaining of visual strain, headache and physical discomfort. We found nearly maximal satisfaction with 3D laparoscopy among both attendings and residents. Residents were actually significantly more satisfied with 3D. The study by Hanna et al. was carried out in 1998 and laparoscopic instrumentation has evolved since. Newer studies with high-definition 3D laparoscopes have not reported surgeon discomfort during 3D laparoscopy [7, 8].

A recent systematic review evaluating 3D laparoscopy in general included thirteen randomized controlled studies, of which only two were clinical trials including a total of 162 patients [5]. One of these trials assessed 3D in vesicoureteral anastomosis and found no difference in regards to time to complete anastomosis compared to 2D laparoscopy [7]. Another trial reported decrease of operative time in mini-gastric bypass, but not in sleeve gastrectomy when using 3D compared to 2D laparoscopy [9]. Most of the studies (eleven) in the systematic review were experimental, in which 3D was either equal or superior to 2D laparoscopy. The systematic review concluded that these findings need to be validated in larger clinical trials.

A few randomized trials assessing 3D in LCC have been published. Komaei et al. [10] conducted a systematic review and found only five randomized controlled trials comparing 2D to 3D in LCC reporting all together 209 patients [10]. Three of these trials reported a significant reduction in operative time in 3D LCC [9, 11, 12], while two did not find such difference [4, 13]. The systematic review concluded that bigger cohort sizes are needed to assess 3D laparoscopy and justify the increased costs of new, more expensive 3D systems.

This study has limitations. First, the study was not blinded from the surgeons’ side as this would have been impossible given the nature of the study. Second, no validated questionnaire to assess surgeons’ fatigue, nausea, or distress was used. The surgeons scaled their satisfaction to the laparoscope on a Likert scale and were free to report any complaints as free text. Third, the time spent performing different tasks during the operation were not recorded. Therefore, we do not know whether some parts of the procedure have been faster with 3D. However, our primary outcome, whole operative time, is more practical. Fourth, the primary endpoint was operative time, and one could argue that better endpoints in a clinical trial would have been morbidity and mortality. However, these were included as secondary endpoints, but this study was not powered to detect differences in major complications or mortality, as these are very rare events in elective LCC. There were no hints that these would be altered by the 3D or 2D approach.

The strength of this study is large sample size. Large numbers of patients and surgeons were involved with various levels of expertise allowing a subgroup analyses to be performed. The study designs were rather pragmatic, and as such, the results are highly applicable to daily practice.

In our study, three-dimensional laparoscopy does not improve surgical efficacy of elective LCC. There were no differences between 3D and 2D in terms of surgical safety, and it seems unlikely that any clinically relevant differences will be found even if larger trials would be carried out.
